# *Actinomyces* and *Shewanella algae* complicated paediatric mastoiditis: a case report of a multidisciplinary approach

**DOI:** 10.1099/acmi.0.000436

**Published:** 2022-12-05

**Authors:** Mafalda Martins Sousa, Madalena von Hafe, Ana Reis-Melo, Helena Silveira, Gil Coutinho, Carla Pinto Moura

**Affiliations:** ^1^​ Department of Otorhinolaryngology, Centro Hospitalar Universitário São João, EPE, Porto, Portugal; ^2^​ Unit of Otorhinolaryngology, Departament of Surgery and Physiology, Faculty of Medicine, University of Porto, Porto, Portugal; ^3^​ Department of Pediatrics, Centro Hospitalar São João EPE, Porto, Portugal; ^4^​ Department of Surgery and Physiology, Faculty of Medicine, University of Porto, Porto, Portugal; ^5^​ Pediatric Infectious Diseases and Primary Immunodeficiencies Unit, Centro Hospitalar Universitário do São João, Porto, Portugal; ^6^​ Genetics Department, Department of Pathology, Faculty of Medicine, University of Porto, Porto, Portugal; ^7^​ Institute for Research and Innovation in Health/Instituto de Investigação e Inovação em Saúde i3S, Portugal, Porto University, Porto

**Keywords:** actinomycosis, acute otitis media, mastoiditis, *Shewanella*

## Abstract

Cerebral venous sinus thrombosis in children is a rare complication of acute mastoiditis that can potentially be fatal. Clinical expertise is essential for early diagnosis and management due to its subtle course. We present the first known case of paediatric acute mastoiditis with venous sinus thrombosis caused by *Shewanella algae* and *Actinomyces europaeus *.

A 17-year-old male presented clinical signs of right acute otitis media and mastoiditis. Brain computed tomography showed mastoid opacification, cerebral sinus thrombosis and an extradural collection. Microbiology revealed the presence of *S. algae* and *A. europaeus *. A multidisciplinary approach combining medical and surgical treatment allowed the patient to make a full recovery.

## Introduction

Acute otitis media (AOM) is one of the most common paediatric infectious diseases. Although suppurative complications have become rare due to improvements in therapeutic approaches, possible serious complications might occur. The most common AOM complication is acute mastoiditis (AM) [1]. Intracranial complications in patients with acute mastoiditis can occur in up to 3 % of cases [2]. Regarding aetiology, *Streptococcus pneumoniae* is the most common pathogen in children with AM and is also the one most frequently associated with complications. Other pathogens commonly identified are *Streptococcus pyogenes *, *Staphylococcus aureus* and *Haemophilus influenzae. Pseudomonas aeruginosa* also plays a role, especially in older children and in acute mastoiditis resulting from recurrent or chronic otitis media [3]. *Actinomyces* are Gram-positive, anaerobic non-acid-fast and filamentous bacteria. *Actinomyces* are commensal species in humans and are frequently found in the oropharynx and genitourinary tract [4]. Infections with *Actinomyces* spp., despite being rare, do occur more frequently in the orocervicofacial region. There are few reports in the literature of middle ear and mastoid commitment. [4]. *Shewanella* spp. are emerging human pathogens, with *Shewanella algae* being the leading species. *Shewanella* skin and soft tissue infections are more usually seen in immunocompromised patients with a preexisting cutaneous ulcer and are most commonly associated with exposure to marine environments [5]. For *S. algae *, direct contact with seawater or ingestion of contaminated seafood are established risk factors for infections [6].

## Case presentation

A previously healthy and fully immunized 17-year-old male patient was admitted to the paediatric emergency department with persistent earache and otorrhea, recently associated with retroauricular pain. One month before, he had been on oral amoxicillin/clavulanate and topical ofloxacin for 10 days to treat right acute otitis media, without any improvement. He returned to the paediatric emergency department with persistent symptoms. At that time, ceftriaxone 1 g was prescribed for 3 days, but with no modification of the clinical signs and symptoms. Clinical examination showed right otorrhea, retroauricular swelling, redness and pain on palpation of the mastoid. Otoscopy was consistent with right acute otitis media.

Neurological impairment or meningeal involvement were not present. Laboratory tests revealed haemoglobin of 16.1 g dl^−1^ [normal range (*N*): 13.8–17.2 g dl^−1^], white blood cell count of 14.15×10^9^ μ^−1^l (*N*: 4–11×10^9^ μl^−1^), with neutrophil predominance and an elevated C-reactive protein (CRP) of 76.7 mg l^−1^ (*N*<3 mg l^−1^). Blood culture was performed and was negative. The patient declared not having attended swimming pools, the sea or rivers. Additionally, there was a family history of recurrent ear infections in his father and grandmother.

**Fig. 1.**
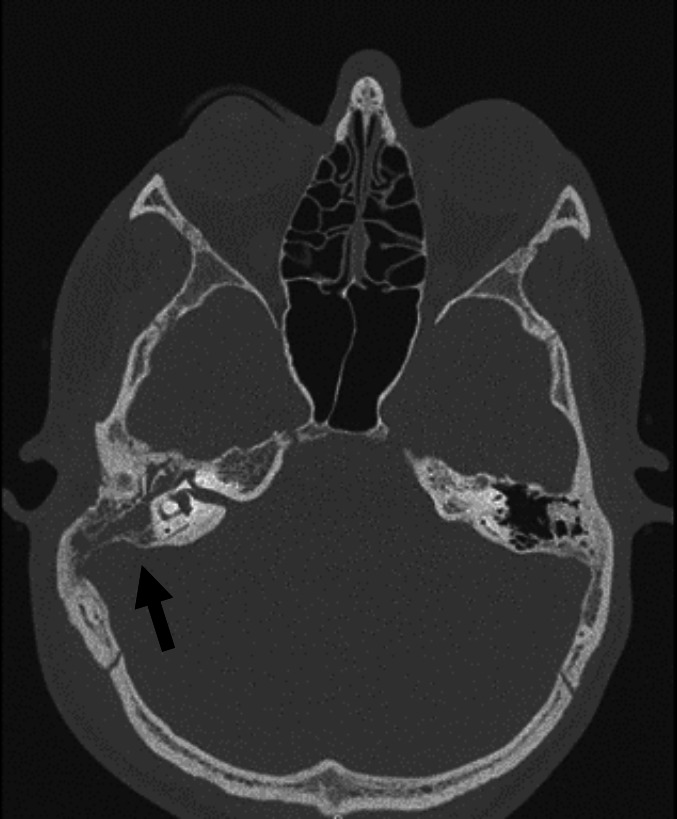
Computed tomography showing obliteration of the mastoid and tympanic cavity with ossicular erosion compatible with otomastoiditis.

**Fig. 2.**
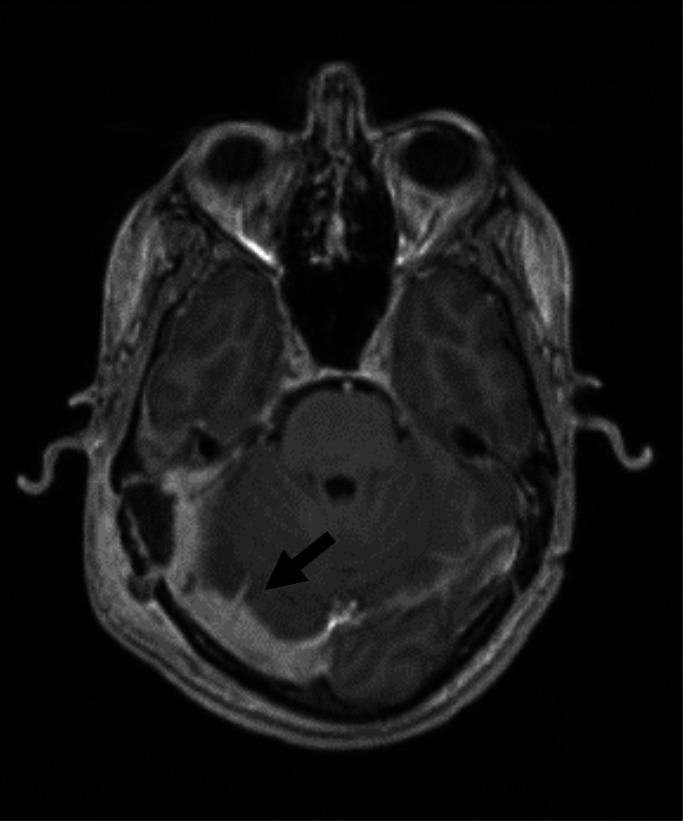
Magnetic resonance image (T1-weighted sequence): Thrombosis of right sigmoid sinus. Inflammatory thickening of dura-mater with involvement of right temporal basal region. Oedema and contrast enhancement of retroauricular and occipital soft tissues.

A contrast-enhanced computed tomography (CT) scan showed right mastoid opacification, swelling of adjacent soft tissues, ossicular erosion, extension of the infection to the extradural space and partial thrombosis of the right transverse sinus (Fig. 1). Magnetic resonance imaging (MRI) was also performed and revealed signs of acute mastoiditis with intracranial empyema in the transverse and sigmoid sinus and there was no evidence of involvement of the brain parenchyma (Fig. 2). Empirical antibiotic treatment was started immediately with vancomycin intravenous (IV) (350 mg, 6/6 h), ceftriaxone IV (2 g, 12/12 h) and metronidazole IV (375 mg, 6/6 h).

The patient underwent a right canal wall-up mastoidectomy, with skeletonization of the cortical bone for sinus management and myringotomy with placement of a Goode T-ventilation tube.

The bacterial culture of the purulent drainage obtained during surgery was positive for *S. alga* e and *Actinomyces europaeus,* using a chocolate agar culture and the matrix-assisted laser desorption/ionization time-of-flight (MALDI-TOF) technique*,* which allowed an antibiotic adjustment after knowledge of bacterial isolation. *S. alga* e was sensitive to ceftazidime (inhibition zone 6.5–27.5 mm) [7], amikacin and trimethoprim/sulfamethoxazole, had intermediate sensitivity to ciprofloxacin and was resistant to imipenem [according to the European Committee for Antimicrobial Susceptibility Testing (EUCAST) criteria). Of note, it was not possible to perform the antibiotic sensitivity test for *A. europaeus *, since there are no EUCAST reference values.

Histopathology evaluation revealed oedematous fibrovascular tissue with marked acute inflammation. Granulation tissue, sulfur granules and neutrophils were present within the inflamed stroma. The tests for micro-organisms using periodic acid–Schiff stain, Grocoot and Gram were negative.

The antibiotic regimen was then changed to ceftazidime to cover *S. alga* e and penicillin G to inhibit *Actinomyces *. The total duration of intravenous antibiotic treatment was 4 weeks. Ofloxacin drops three times a day were also applied. Considering the sinus thrombosis, subcutaneous low-molecular-weight heparin (LMWH) 1 mg/kg/dose twice a day was initiated on the day after the surgery and later switched to warfarin.

The patient remained apyretic and haemodynamically stable throughout the hospital stay. The hospitalization was uneventful. Gradual normalization of the white blood cell count and CRP were documented. MRI was performed again 15 days after surgery, revealing an improvement in the appearance of mastoid inflammation and soft tissue swelling. Partial recanalization of the right sigmoid and transverse sinus was also evidenced. After 4 weeks the patient was discharged in good general condition and continued anticoagulation for 6 months and oral amoxicillin (1.5 g/day) for at least 6 other months. In the follow-up visits, immunodeficiency was ruled out. After 8 months he had mild conduction hearing loss, with no other symptoms.

## Discussion

Otogenic suppurative complications are rare in paediatric patients but might have a high morbidity and mortality if not expeditiously diagnosed and treated [8]. Patients diagnosed with acute mastoiditis are at an increased risk of intracranial involvement because of the proximity of the sigmoid sinus to the mastoid. The inflammation and oedema increase vascular pressure, which consequently predisposes patients to venous stasis and hypercoagulability. Although cerebral venous sinus thrombosis typically presents with high-grade fever, otalgia, otorrhea and altered mental status, previous use of antibiotics may result in a subtle presentation, as in this case report.

A 2020 literature review showed that most cases of paediatric otogenic cerebral venous sinus thrombosis have negative bacterial culture tests of the ear exsudate [9]. Although there are some cases of ear infections caused by *S. alga* e, to our knowledge, this is the first published case of acute mastoiditis due to this agent. *S. algae* is a rod-shaped, Gram-negative marine bacterium often found in aquatic environments. It is the infectious agent in multiple clinical cases due to the ingestion of raw seafood [5]. *Shewanella* infections have frequently been associated with coastal areas and warm climates [5].

Our patient denied having attended aquatic sites or ingestion of raw seafood [6]. This finding is in line with current evidence that shows that a number of patients with infection by *S. algae* have not recently been exposed to marine environments or products. As the source of infection is often unknown, it seems that *Shewanella* spp. might have a more widespread distribution, and frequently be associated with other pathogenic microbes [10].

*Shewanella* is sensitive to many antibiotics, namely aminoglycosides, fluoroquinolones, extended-spectrum cephalosporins, β-lactamase inhibitors, carbapenems, macrolides, aztreonam and trimethoprim/sulfamethoxazole [5]. The emergence of resistant strains is worrying and increasing; the mechanism to this resistance is the presence of qnrA genes [11].

Reports of *S. algae* infections in human have been increasing in recent years, with one study reporting detection of *Shewanella* spp. in 63 ear infections in young patients with no comorbidities [12]. To the best of our knowledge, there is only one other report of acute mastoiditis caused by *A. europaeus* in a paediatric patient [13].

Actinomycosis is a rare disease caused by Gram-positive bacteria of the family *Actinomycetaceae *. *A. europaeus* was first described in 1997 as a new species causing mainly skin and soft tissue infections. It has been isolated not only in skin and soft tissue infections, but also in urinary tract infections, most often in polymicrobial culture, and is usually considered to be nonpathogenic commensal flora [14]. *Actinomyces* species are also commensal flora of the oral cavity and nasopharynx, so it is possible that, in this case, the Eustachian tube was the route of infection to the middle ear. Infections directly from the external auditory canal or through haematogenous spread are less probable alternatives [13].

The diagnosis of actinomycosis requires the fulfilment of two out of three of the following criteria: positive culture, positive histopathology or sulfur granules in the pus [4]. Of note, cultures for *Actinomyces* are negative in ~50 % of cases, and cultures should be followed for an extended duration of time. It is mainly a disease of adults and also occurs in those in immunocompromises states [14].

In our case, we had a positive culture, susceptible *in vitro* to a wide range of beta-lactam antibiotics and the presence of sulfur granules in the pus. Treatment must comprise surgery to remove necrotic tissue and a prolonged (6 to 12 month) course of high doses of penicillin G or amoxicillin. However, the length of antimicrobial therapy could probably be shortened to 3 months in patients who have undergone optimal surgical resection of infected tissues [15]. Drug resistance is not regarded as a problem in actinomycosis. However, it is important to note that *A. europaeus,* along with other species such as *Actinomyces graevenitzii *, are resistant to ceftriaxone [16]. Physicians should be aware of the different clinical forms of actinomycosis and suspicion is essential for an accurate diagnosis, either by using specific culture media that allow the growth of *Actinomyces* spp. or by looking for sulfur granules [16].

Treatment duration for acute mastoiditis with venous sinus thrombosis is still uncertain, and more studies are needed to establish guidelines. Anticoagulation therapy and surgical treatment in otogenic cerebral venous sinus thrombosis are still being discussed. Anticoagulation seems beneficial in restricting the cerebral thrombus, improving intracranial drainage and preventing a rise in intracranial pressure. The duration of hypocoagulation is also a topic of debate, but 6 months seems to be a safe approach. Studies show a 52 % partial or complete venous recanalization in children with otogenic sinous thrombosis [17]. It is always important to analyse the benefits of hypocoagulation against the side effects, such as induced thrombocytopenia, bleeding or the release of septic emboli. Regarding surgery, the current trend is to perform a mastoidectomy with removal of inflammatory tissue from the sinus walls to eliminate the perisinus infection. More aggressive procedures, such as surgical sinus drainage with removal of the thrombus, are not routinely advocated [9]. Treatment should always be multidisciplinary and surgery is of central importance. A long-term follow-up of the patient is recommended to avoid and detect recurrences.

## Conclusion

Otogenic complications are rare but still occur in paediatric patients. Clinicians should be alert to this condition as clinical presentation might be subtle. Early diagnosis is important for a good outcome and microbiology had an important role in our case and allowed targeted treatment.

*Actinomyces* and other rare agents such as *Shewanella* spp. should be considered to be one of the differential causes in patients with no improvement after medical treatment. Combined medical and surgical treatment is the recommended management, with long-term antibiotic therapy. In cases of sinus thrombosis, concomitant anticoagulants might increase the recanalization rate.

## References

[1] Schilder AGM, Marom T, Bhutta MF, Casselbrant ML, Coates H (2017). Panel 7: Otitis Media: treatment and complications. Otolaryngol Head Neck Surg.

[2] Wong BYW, Hickman S, Richards M, Jassar P, Wilson T (2015). Management of paediatric otogenic cerebral venous sinus thrombosis: a systematic review. Clin Otolaryngol.

[3] Cassano P, Ciprandi G, Passali D (2020). Acute mastoiditis in children. Acta Biomed.

[4] Gellman SR, Milera A, DeNapoli TS, Buckmiller LM, Castagnini LA (2019). Actinomyces mastoiditis in a 5-year-old male. Clin Pediatr.

[5] Tseng S-Y, Liu P-Y, Lee Y-H, Wu Z-Y, Huang C-C (2018). The pathogenicity of *Shewanella algae* and ability to tolerate a wide range of temperatures and salinities. Can J Infect Dis Med Microbiol.

[6] Ignak S, Unay Demirel O, Soydan S, Esen E (2018). *Shewanella algae* in a chronic suppurative otitis media patient with cholesteatoma. DD&T.

[7] Yang X, Wang D, Zhou Q, Nie F, Du H (2019). Antimicrobial susceptibility testing of *Enterobacteriaceae*: determination of disk content and Kirby-Bauer breakpoint for ceftazidime/avibactam. BMC Microbiol.

[8] Zanoletti E, Cazzador D, Faccioli C, Sari M, Bovo R (2015). Intracranial venous sinus thrombosis as a complication of otitis media in children: critical review of diagnosis and management. Int J Pediatr Otorhinolaryngol.

[9] Castellazzi ML, di Pietro GM, Gaffuri M, Torretta S, Conte G (2020). Pediatric otogenic cerebral venous sinus thrombosis: a case report and a literature review. Ital J Pediatr.

[10] Torri A, Bertini S, Schiavone P, Congestrì F, Matteucci M (2018). *Shewanella algae* infection in Italy: report of 3 years’ evaluation along the coast of the northern Adriatic Sea. New Microbes New Infect.

[11] Kim HB, Park CH, Gavin M, Jacoby GA, Hooper DC (2011). Cold shock induces *qnrA* expression in *Shewanella algae*. Antimicrob Agents Chemother.

[12] Holt HM, Søgaard P, Gahrn-Hansen B (1997). Ear infections with *Shewanella alga*: a bacteriologic, clinical and epidemiologic study of 67 cases. Clin Microbiol Infect.

[13] Coutinho G, Spratley J, Saldanha I, Castro C, Pinheiro J (2020). Middle ear actinomycosis. J Pediatr Infect Dis.

[14] Funke G, Alvarez N, Pascual C, Falsen E, Akervall E (1997). *Actinomyces europaeus* sp. nov., isolated from human clinical specimens. Int J Syst Bacteriol.

[15] Fernández LV, Arias E, Cohen D, Spini R (2021). Actinomycosis in temporal bone. A pediatric case report. Arch Argent Pediatr.

[16] Wong VK, Turmezei TD, Weston VC (2011). Actinomycosis. BMJ.

[17] Novoa E, Podvinec M, Angst R, Gürtler N (2013). Paediatric otogenic lateral sinus thrombosis: therapeutic management, outcome and thrombophilic evaluation. Int J Pediatr Otorhinolaryngol.

